# Impact of Leaflet Tethering on Residual Regurgitation in Patients With Degenerative Mitral Disease After Interventional Edge-to-Edge Repair

**DOI:** 10.3389/fcvm.2021.647701

**Published:** 2021-04-29

**Authors:** Zhenyi Ge, Wenzhi Pan, Wei Li, Lai Wei, Dehong Kong, Cuizhen Pan, Daxin Zhou, Xianhong Shu, Junbo Ge

**Affiliations:** ^1^Shanghai Institute of Medical Imaging, Fudan University, Shanghai, China; ^2^Department of Echocardiography, Zhongshan Hospital, Fudan University, Shanghai, China; ^3^Shanghai Institute of Cardiovascular Disease, Fudan University, Shanghai, China; ^4^Department of Cardiology, Zhongshan Hospital, Fudan University, Shanghai, China; ^5^Department of Cardiac Surgery, Zhongshan Hospital, Fudan University, Shanghai, China

**Keywords:** 3D transesophageal echocardiography, degenerative mitral regurgitation, mitral valve geometry, interventional edge-to-edge repair, minimally invasive surgery

## Abstract

**Background:** Grade 2+ residual mitral regurgitation (MR 2+) is associated with the recurrence of MR and a lower survival rate in interventional mitral valve (MV) edge-to-edge (EE) repair. We sought to determine the MV anatomic factors affecting residual MR 2+ during interventional EE repair with the ValveClamp system in patients with degenerative MR (DMR).

**Methods:** In this multicenter study, 62 patients with significant (grade 3+ to 4+) DMR underwent ValveClamp implantation across eight centers from July 2018 to December 2019. Patient clinical, anatomical, and procedural characteristics were prospectively collected and retrospectively analyzed.

**Results:** A single clamp was implanted in 59 patients, and two clamps were implanted in three patients. Residual MR 2+ was found in 14 patients (22.6%) immediately after the ValveClamp procedure. Patients with residual MR 2+ showed significantly larger preoperative tenting sizes and annular dimensions than the residual MR ≤1+ group. Multivariate analysis identified tenting volume as the major determinant of residual MR 2+ after ValveClamp procedures (odds ratio, 1.410 per 0.1-mL/m^2^ increase; 95% confidence interval, 1.167–1.705; *P* < 0.001). Receiver operating characteristic curves identified a tenting volume index ≥0.82 mL/m^2^ as the optimal cutoff point to predict residual MR 2+ (area under curve, 0.84). Patients with a tenting volume index ≥0.82 mL/m^2^ were more likely to develop recurrent 3+ MR or undergo MV surgery during short-term follow-up (*P* < 0.001).

**Conclusions:** Preoperative assessment of the tenting volume index will help to predict intraoperative residual MR 2+ in patients with DMR receiving EE-based interventional repair. Improvements in the interventional strategy are warranted for sustained MR reduction in patients with DMR with unfavorable anatomy.

## Introduction

Interventional mitral valve (MV) edge-to-edge (EE) repair using the MitraClip system has changed the landscape for the treatment of symptomatic severe mitral regurgitation (MR) (1). Previous studies have revealed that residual MR ≥2+ after MitraClip implantation is associated with the recurrence of MR and a lower survival rate in degenerative MR (DMR) (2, 3). DMR has a heterogeneous pathophysiological spectrum and highly variable anatomy; however, the specific group of patients who would benefit from this therapy remains to be determined (4, 5).

The advent of quantitative three-dimensional (3D) echocardiography and MV modeling has improved our understanding of the morphological distortion within DMR (6–8). Recently, it has been suggested that the preoperative 3D echocardiography-derived annular diameter and tenting/tethering size can predict the response to MitraClip implantation in patients with DMR. However, these studies were limited by mixed or limited study populations, and the results were discordant (9–11).

The ValveClamp system (Hanyu Medical Technology, Shanghai, China) is a transapical MV EE repair device designed for ease of operation. The first-in-human study of the ValveClamp system demonstrated its feasibility and safety in a patient cohort with severe DMR (12).

In this multicenter study, we sought to further determine the preoperative MV anatomic determinants of residual MR 2+ after interventional EE repair with the ValveClamp system among patients with DMR.

## Materials and Methods

### Patients

From July 2018 to December 2019, consecutive patients with DMR who underwent ValveClamp procedures across eight tertiary centers in China were included in the analysis. All patients underwent transthoracic and transesophageal echocardiography (TEE) and were evaluated by a multidisciplinary heart team.

The inclusion criteria for ValveClamp procedures were as follows: (a) age older than 60 years, (b) moderate to severe (3+) or severe (4+) DMR, (c) symptoms [New York Heart Association (NYHA) cardiac function class ≥2] related to MR, (d) primary regurgitant jet originated from malcoaptation of the A2 and P2 scallops, (e) high risk for surgery according the Mitral Valve Academic Research Consortium criteria, and (f) provision of signed informed consent. The exclusion criteria for ValveClamp procedures were as follows: (a) significant regurgitation beyond A2/P2 scallops, (b) rheumatic disease or MV perforation, (c) leaflet calcification of the grasping zone, (d) acute endocarditis, (e) coronary artery disease requiring revascularization, (f) significant concomitant non-MV disease, (g) life expectancy <12 months, and (h) recent cerebral event (12, 13).

Sixty-four patients who fulfilled the inclusion criteria were initially enrolled to receive ValveClamp implantation. Two patients experienced failure of ValveClamp implantation (one developed an MV leaflet tear, and the other had a severe residual MR) and were converted to open heart surgery. The remaining 62 patients were included in the study. The study was approved by the local institutional review board at each participating institution and was conducted in accordance with the Declaration of Helsinki.

### ValveClamp Procedures

**Figure 1:**
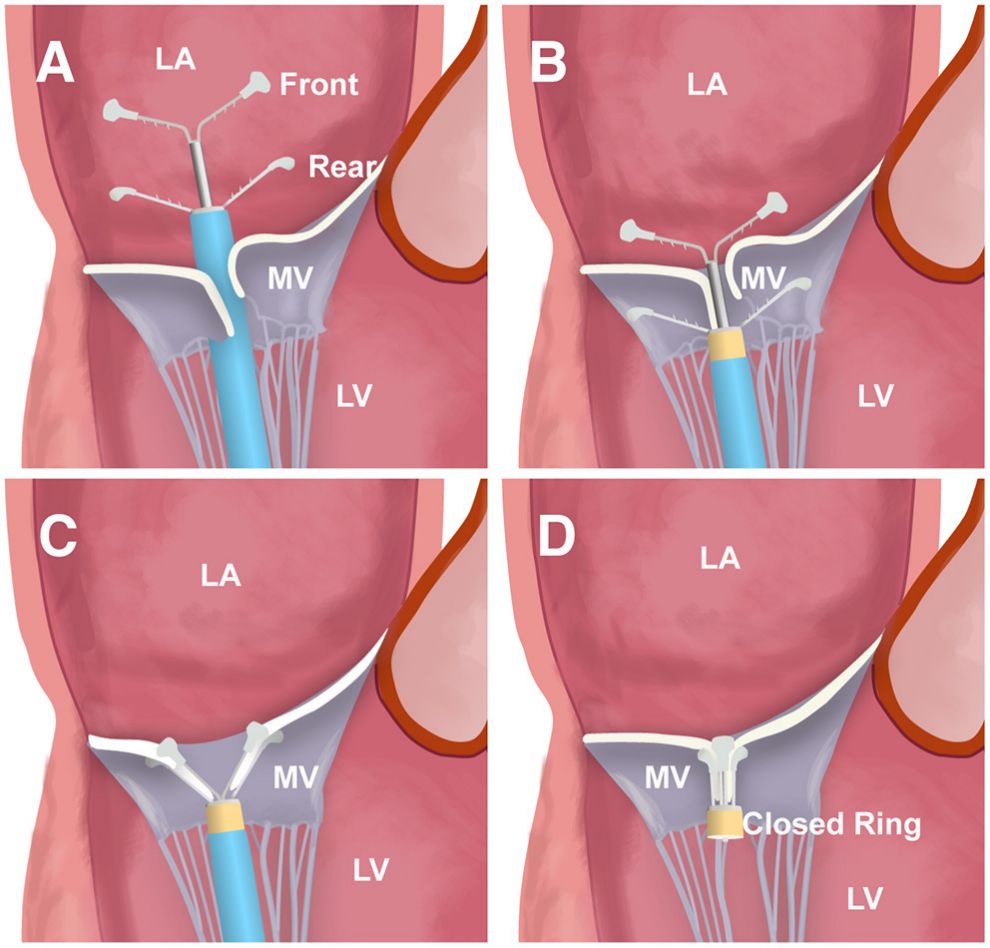
The main steps of ValveClamp implantation. **(A)** The clamp is delivered and opened within the left atrium (LA). **(B)** The clamp is adjusted to the appropriate position; the rear clamp is placed just under the leaflets, and the front clamp remains within the LA. **(C)** The front clamp is pulled back to capture the leaflets, and then the closed ring is moved forward to cover the ventricular end of the clamp arms, approximating the mitral valve (MV) leaflets and strengthening the clamp. **(D)** The clamp is released.

The ValveClamp system was designed as a transapical means to reduce MR by approximating the MV leaflets. Transapical ValveClamp implantation involves the introduction of a leaflet clamp into the left atrium (LA) and coaxial deployment of the clamp to the MV (Figure 1). The clamp is composed of a front clamp, rear clamp, and closed ring. The arm lengths of the front and rear clamps were 9 and 10 mm, respectively. The configuration of the clamp arms was designed to expand the capture range for grasping leaflets, and it was considerably larger than that of the MitraClip, despite the similar grasping arm dimensions (Figure 1). The implantation procedures were performed as described previously (12, 13). Transthoracic echocardiography (TTE) was used to identify the optimal location for an incision to expose the cardiac apex. Biplane TEE imaging, with a standard bicommissural two-chamber view and orthogonal long-axis view, was employed to facilitate the transapical puncture. A 16F introducer sheath was introduced and advanced into the LA using a valve-crossing device, and the clamp was then delivered to the LA via the introducer sheath. Next, the front and rear clamps were opened within the LA under TEE monitoring. A combination of MV 3D face view and biplane imaging was used to position the clamp arms toward the middle of the regurgitant jet and perpendicular to the MV coaptation line. After checking its position and orientation, the rear clamp was gently retrieved back into the left ventricle (LV) to hold the MV leaflets. The front clamp was pulled back to grasp the leaflets, and then the closed ring was moved forward to approximate the MV leaflets and strengthen the clamp. Prior to release, the length of leaflet insertion, severity of residual MR, and degree of stenosis were assessed. Additional clamps may be placed to achieve further MR reduction if needed.

### Echocardiography

All patients underwent TTE at baseline and before discharge. The LV end-diastolic volume (EDV), LV end-systolic volume (ESV), and LV ejection fraction (LVEF) were measured using the biplane method of disks in the apical 2- and 4-chamber views. The systolic pulmonary artery pressure (sPAP) was measured in accordance with the current guidelines (14). The MV area was measured using the 3D planimetry method. MR severity was assessed by the core laboratory echocardiographer using a multiparametric approach according to the current American Society of Echocardiography guidelines (15). Residual MR 2+ immediately after ValveClamp implantation was defined as at least one 2+ regurgitant jet [determined by flow convergence, vena contracta width (VCW) and vena contracta area (VCA)] or a total VCA for multiple residual jets ≥0.2 cm^2^ (16). The etiology of DMR was classified as Barlow disease or fibroelastic deficiency according to the prolapse volume (6).

The preprocedural and postprocedural assessments were performed using intraprocedural TEE imaging with the patient under general anesthesia. Preprocedural MR assessment typically occurred before the transapical puncture, and postprocedural assessment was performed immediately after deployment.

Three-dimensional TEE datasets were acquired as part of the routine perioperative TEE assessment using a Philips EPIQ system (Philips Medical Systems, Andover, MA) with an X7-2t or X8-2t TEE probe. The probe was positioned at the midesophageal level to acquire focused volume datasets encompassing the entire MV and mitral annulus before and immediately after ValveClamp implantation using single-beat 3D zoom mode or 4-beat full-volume mode. In patients with atrial fibrillation, a 3D zoom mode with single-beat volume acquisition was performed to avoid stitch artifacts.

### MV Navigation Analysis

Imaging datasets were analyzed by the core laboratory echocardiographer using Philips Mitral Valve Navigation (MVN) software (QLAB version 10.8; Philips). Images with the highest volume rate (≥10 Hz) and the best image quality were selected for preprocedural and postprocedural analyses. The MVN software provides semiautomated 3D modeling and quantification of the mitral annulus and apparatus. Measurements were performed by two core laboratory echocardiographers who were blinded to the results of the procedure. The mean of three measurements of each parameter was calculated and reported as the final value.

MVN analysis was performed as described previously (13). The end-systolic frame was identified as the last systolic frame just before aortic valve closure and was selected to perform MVN analysis. To define the annulus geometry, we assessed the anterior–posterior (AP) diameters; lateral/medial (ALPM) diameters; annulus height, area, and circumference; and MV annular ellipticity (defined as the ALPM/AP diameter).

Prolapse height and volume were assessed for the quantification of MV prolapse severity. Moreover, the tenting height and volume and the anterior mitral leaflet (AML) and posterior mitral leaflet (PML) angles were assessed for the quantification of MV leaflet tethering. In addition, the total leaflet area was obtained from the end-diastolic frame MVN analysis (the frame before the leaflets were coapted). The leaf-to-annulus area ratio was calculated as the total leaflet area/annular area (17).

Tenting volume and prolapse volume were defined as the volume enclosed between the non-planar mitral annulus and the tethered segments (bulging toward the LV apex) or the prolapse segments (bulging toward LA) of the MV leaflets in 3D space, respectively. The tenting volume was equal to the sum of the AML and PML tenting volumes. Tenting height and prolapse height were defined as the perpendicular distance between the non-planar mitral annulus and the lowest point of tethered segments or the highest point of the prolapsing segments, respectively.

The reproducibility of the preoperative MVN variables was assessed in 15 randomly selected patients. Intraobserver variability was assessed using repeated measurements, and interobserver variability was evaluated by repeated analysis by a second independent observer. Variability was expressed in terms of the coefficients of variation between repeated measurements using the intraclass correlation coefficient (ICC).

### Study Endpoint

After ValveClamp implantation, patients were followed up at the outpatient clinic of each center. We determined the freedom of recurrent MR 3+ or MV surgery after ValveClamp implantation with a 1-year follow-up period.

### Statistical Analysis

The clinical, anatomic, and procedural characteristics of patients with residual MR ≤1+ were compared with those of the residual MR 2+ group. The normality of continuous data was analyzed using the Kolmogorov–Smirnov test. Continuous variables are reported as mean ± SD, and categorical variables are reported as proportions. Differences were detected using the independent Student *t*-test for normally distributed continuous variables, Wilcoxon rank sum test for non-parametric variables, and χ^2^ or Fisher exact test for categorical variables, depending on sample size. A paired *t*-test was used to test the differences between preprocedural and postprocedural 3D TEE measurements within the two groups. The association between 3D TEE anatomic characteristics was investigated using linear regression analysis. Univariate and multivariate predictors of residual MR 2+ MR reduction were identified using logistic regression analysis. Multicollinearity was tested using the variance inflation factor. A receiver operating characteristic (ROC) curve was drawn to identify the cutoff value for residual MR 2+. The Kaplan–Meier method was used to estimate the freedom of recurrent grade 3+ MR or MV surgery at 1 year, and a log-rank test was performed to compare the distribution of free times between groups. All statistical tests were two-tailed, and *P* < 0.05 was considered to indicate statistical significance.

## Results

### Patient Characteristics and Postprocedural Outcomes

The baseline clinical characteristics of the patients are shown in Table 1. The mean age of the patients was 74.67 ± 5.79 years, and 61% of the cohort were women. There were high rates of concurrent comorbidities (mean Society for Thoracic Surgeons risk score, 8.22 ± 2.60%), and the majority of patients (82%) had NYHA functional class III or IV symptoms before the procedure.

**Table 1:** Baseline clinical characteristics.

**Parameters**	**Total (*n* = 62)**	**Residual MR ≤+ (*n* = 48)**	**Residual MR 2+ (*n* = 14)**	***P*-value**
Age (y)	74.67 ± 5.79	74.00 ± 5.75	75.07 ± 6.96	0.561
Sex, female, *n* (%)	38 (61)	31 (65)	7 (50)	0.324
BSA (m^2^)	1.54 ± 0.16	1.52 ± 0.16	1.55 ± 0.15	0.559
Hypertension, *n* (%)	26 (42)	23 (48)	3 (21)	0.077
Atrial fibrillation, *n* (%)	19 (31)	15 (31)	4 (29)	1.000
CAD, *n* (%)	13 (21)	12 (25)	1 (7)	0.284
COPD, *n* (%)	12 (19)	10 (21)	2 (14)	0.872
NYHA III or IV, *n* (%)	52 (84)	39 (81)	13 (93)	0.531
STS score, *n* (%)	7.58 ± 2.60	7.46 ± 2.21	7.97 ± 3.72	0.523
NT-proBNP (pg/mL)	1,572.05 ± 2,029.74	1,098.20 ± 1,496.69	2,065.86 ± 3,055.78	0.095
Creatinine (mg/dL)	83.15 ± 26.3	78.11 ± 24.39	103.27 ± 24.89	0.004

*CAD, coronary artery disease; COPD, chronic obstructive pulmonary disease; NYHA, New York Heart Association functional class; STS, Society for Thoracic Surgeons risk score; NT-proBNP, N-terminal pro–brain natriuretic peptide*.

There was no procedural or in-hospital mortality. A single clamp was implanted in 59 patients (95.2%), and two clamps were implanted in three patients (4.8%). Immediately after ValveClamp implantation, MR was graded as none or trivial in 22 (35%), mild in 26 (42%), moderate in 12 (19%), and moderate to severe in 2 (3%) patients. Accordingly, 48 patients were included in the residual MR ≤1+ group, and 14 were included in the residual MR 2+ group.

### Two-Dimensional Echocardiographic Measures

The baseline MV anatomic features are summarized in Table 2. The residual MR 2+ group showed a larger flail gap (9.11 ± 2.54 vs. 6.90 ± 2.50 mm, *P* = 0.005), but there were no significant differences in the incidence of flail (86 vs. 69%, *P* = 0.362) or prolapse/flail width (12.19 ± 3.19 vs. 11.65 ± 3.07 mm, *P* = 0.565). The LV EDV index (80.01 ± 17.79 vs. 70.22 ± 14.22 mL/m^2^, *P* = 0.039) and ESV index (ESVi) (32.73 ± 9.14 vs. 25.99 ± 8.82 mL/m^2^, *P* = 0.023) were significantly larger in the residual MR 2+ group compared with the residual MR ≤1+ group. However, there was no significant difference in LVEF between the two groups. In addition, the VCW was similar between the two groups (*P* = 0.118). sPAP was significantly higher in the residual MR 2+ group than in the residual MR ≤1+ group (63.64 ± 21.33 vs. 49.58 ± 16.45 mm Hg, *P* = 0.021).

**Table 2:** Anatomic and hemodynamic characteristics.

**Parameters**	**Residual MR ≤1+ (*n* = 48)**	**Residual MR 2+ (*n* = 14)**	***P*-value**
PML prolapse, *n* (%)	38 (79)	12 (86)	0.872
Etiology			0.610
FED, *n* (%)	44 (92)	12 (86)	
BD, *n* (%)	4 (8)	2 (14)	
Flail scallop, *n* (%)	33 (69)	12 (86)	0.362
Flail/coaptation gap (mm)	6.90 ± 2.50	9.11 ± 2.54	0.005
Flail width (mm)	11.65 ± 3.07	12.19 ± 3.19	0.565
EDVi (mL/m^2^)	70.22 ± 14.22	80.01 ± 17.79	0.039
ESVi (mL/m^2^)	25.99 ± 8.82	32.73 ± 9.14	0.023
LVEF (%)	63.10 ± 8.97	59.06 ± 8.5	0.139
Mean gradient (mm Hg)	2.17 ± 1.07	2.54 ± 1.48	0.315
VCW (mm)	9.01 ± 2.48	10.33 ± 2.87	0.118
sPAP (mm Hg)	49.58 ± 16.45	63.64 ± 21.33	0.021
**Mitral annulus geometry**			
ALPM index (mm/m^2^)	23.24 ± 3.12	25.83 ± 3.03	0.013
AP index (mm/m^2^)	21.43 ± 2.88	24.08 ± 2.81	0.006
Annular height index (mm/m^2^)	4.27 ± 1.27	4.52 ± 1.52	0.555
Annular area index (cm^2^/m^2^)	6.21 ± 1.17	8.01 ± 1.37	0.000
Annular circumference index (mm/m^2^)	74.03 ± 8.91	83.02 ± 8.63	0.003
AM angle (°)	107.76 ± 7.70	111.56 ± 6.91	0.127
Ellipticity (%)	109.13 ± 11.49	107.72 ± 9.79	0.699
**Mitral valve geometry**			
AML angle (°)	17.78 ± 6.52	20.32 ± 7.86	0.224
PML angle (°)	25.01 ± 7.85	27.74 ± 7.30	0.250
Tenting height index (mm/m^2^)	2.83 ± 1.34	4.6 ± 2.17	0.018
Tenting volume index (mL/m^2^)	0.5 ± 0.3	1.17 ± 0.67	0.005
Prolapse height index (mm/m^2^)	3.08 ± 1.49	3.49 ± 1.47	0.399
Prolapse volume index (mL/m^2^)	0.3 ± 0.27	0.39 ± 0.32	0.349
Non-planar angle (°)	135.92 ± 12.18	130.5 ± 14.23	0.191
Total leaflet area index (cm^2^/m^2^)	7.05 ± 1.62	8.77 ± 1.84	0.001
Leaflet to annulus area (ratio)	1.16 ± 0.09	1.17 ± 0.07	0.668
Flail gap to tenting (height ratio)	2.00 ± 1.30	1.54 ± 0.66	0.211

*PML, posterior mitral leaflet; FED, fibroelastic deficiency; BD, barlow disease; EDVi, end-diastolic volume index; ESVi, end-systolic volume index; LVEF, left ventricular ejection fraction; VCW, vena contracta width; sPAP, systolic pulmonary artery pressure; ALPM, lateral/medial; AP, anterior–posterior; AML, anterior mitral leaflet; AM, aortic–mitral plane*.

### Quantitative MV Navigator Characteristics

With respect to annular geometry, the indexed AP diameter, annular area, and circumference were significantly larger in patients with residual MR 2+ than in those with residual MR ≤1+ (*P* = 0.006, *P* < 0.001, and *P* = 0.003, respectively).

The tenting volume index (1.17 ± 0.67 vs. 0.5 ± 0.3 mL/m^2^, *P* < 0.001) and tenting height index (4.6 ± 2.17 vs. 2.83 ± 1.34 mm/m^2^, *P* = 0.018) were also increased in patients with residual MR 2+. The total leaflet area was significantly larger in the residual MR 2+ group, and the leaflet–to–annulus area ratio was similar between the two groups.

### Postprocedural Measurements

**Figure 2:**
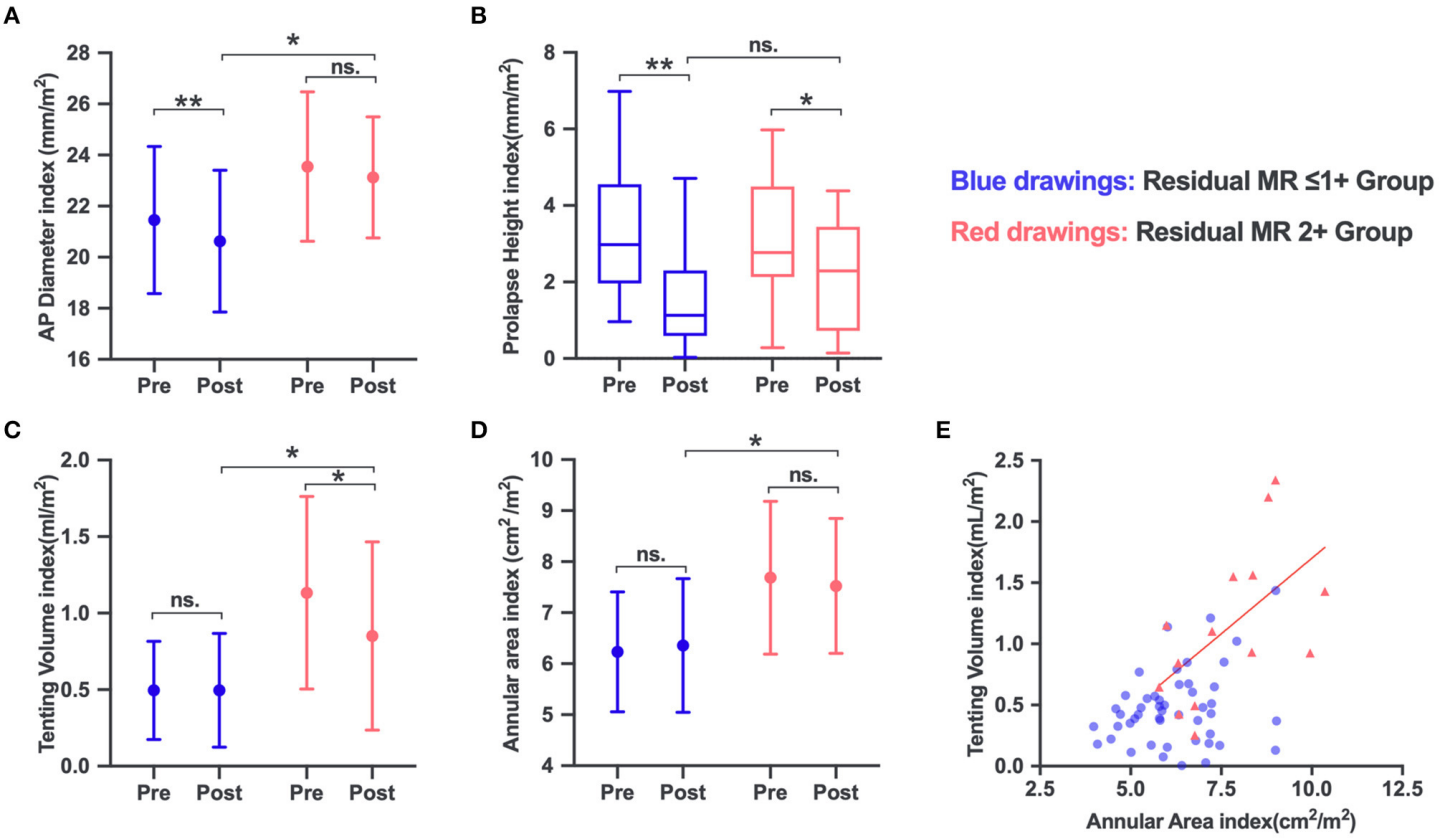
Changes in mitral valve (MV) anatomic parameters in the residual MR 2+ and residual ≤ 1+ groups: AP diameter **(A)**, prolapse height **(B)**, tenting volume **(C)**, and annular area **(D)**. **(E)** The annular area increased in parallel with the tenting volume increase in the residual MR 2+ group, but not in the residual ≤1+ group. ns, no significance. *P < 0.05; **P < 0.001.

Postprocedural MVN analysis was available for 57 patients. A significant reduction in AP diameter (*P* = 0.001) and prolapse height (*P* < 0.001) was observed in the residual MR ≤1+ group. The residual MR 2+ group experienced no obvious reduction in AP diameter (*P* = 0.371), but a significant decrease in tenting volume (*P* = 0.028). The annular area remained unchanged in both groups (*P* = 0.324 for residual MR ≤1+ and *P* = 0.515 for residual MR 2+). As expected, the post-AP diameter index and, importantly, the annular area index and residual tenting volume index in the residual MR 2+ group were significantly larger than those in the residual MR ≤1+ group (*P* = 0.004, 0.006, and 0.013, respectively) (Figure 2).

### Relationship Among MV Anatomic Characteristics

The distribution of the annular area index and tenting volume index is depicted in Figure 2E. The annular area index showed a significant mild correlation with the tenting volume index (*r*^2^ = 0.3, *P* = 0.42) in the residual MR 2+ group. The total leaflet area increased in parallel with the increase in annular area among the groups, and both showed a strong correlation (*r*^2^ = 0.86, *P* < 0.001, for residual MR ≤1+; *r*^2^ = 0.93, *P* < 0.001, for residual MR 2+). Multivariate regression analysis revealed that tenting height (*r*^2^ = 0.170, *P* = 0.001) was associated with the flail gap for the entire series. The flail gap–to–tenting height ratio was balanced between the two groups (2.00 ± 1.30 vs. 1.54 ± 0.66, *P* = 0.211).

### Determinants and Predictors of Residual MR 2+

**Figure 3:**
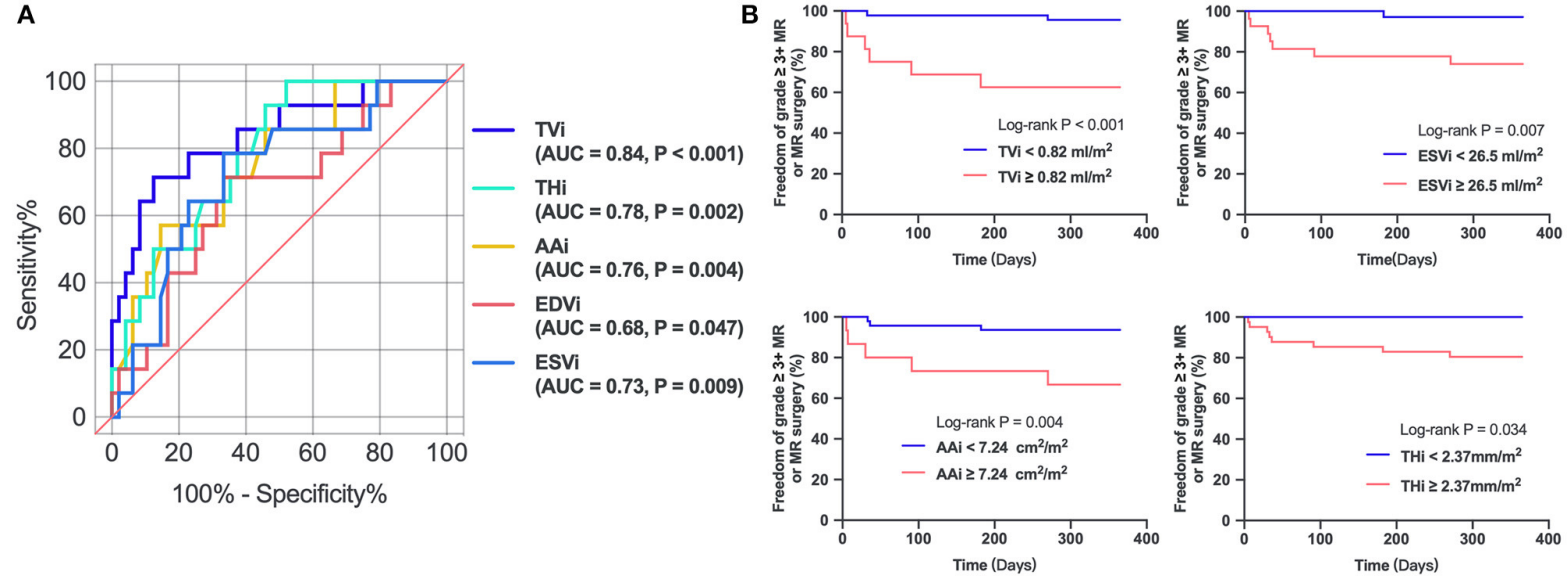
**(A)** Receiver operating characteristic curves of parameters in predicting residual MR 2+. **(B)** Kaplan–Meier estimates freedom from recurrent 3+ MR or MV surgery. TVi, tenting volume index; THi, tenting height index; AAi, annular area index; AUC, area under curve.

In the univariate regression analysis, tenting volume index, tenting height index, annular area index, and ESVi were significantly associated with residual MR 2+ (Table 3). Because of multicollinearity, only the annular area index (not the indexed AP diameter, ALPM diameter, and annular circumference), tenting volume index, tenting height index, and ESVi were entered into the model. Multivariate analysis (Table 3) revealed that only the tenting volume index [odds ratio (OR), 1.410 per 0.1-mL/m^2^ increase; 95% confidence interval (CI), 1.167–1.705; *P* < 0.001] was independently associated with residual MR 2+. ROC analysis (Figure 3) showed that a tenting volume index ≥0.82 mL/m^2^ was the optimal cutoff point for predicting residual MR 2+ (area under curve, 0.839; 95% CI, 0.715–0.963; *P* < 0.001).

**Table 3:** Univariate and multivariate logistic regression analysis of anatomic determinants of residual MR 2+.

	**Univariate**				***P*-value for multivariate**	**Cutoff values**
**Parameters (per 1-unit increase)**	***P*-value**	**OR**	**95% CI**			
EDVi (mL/m^2^)	0.051	1.042	1.000	1.084	–	–
ESVi (mL/m^2^)	0.022	1.081	1.011	1.156	0.079	26.5
ALPM index (mm/m^2^)	0.076	1.189	0.982	1.441	–	–
AP index (mm/m^2^)	0.033	0.717	1.020	1.592	–	–
Annular circumference index (mm/m^2^)	0.024	1.086	1.011	1.167	–	–
Annular area index (cm/m^2^)	0.004	2.130	1.271	3.571	0.349	7.24
Tenting height index (mm/m^2^)	0.004	1.932	1.234	3.205	0.216	2.37
Tenting volume index (mL/m^2^)	<0.001	30.284	4.573	200.548	**<0.001**	**0.82**

*Abbreviations are as in Table 2*.

*Bold values denote the significant P-value for multivariate and the optimal cutoff value*.

### Recurrent More Than Moderate MR or Repeat MV Intervention

The 1-year follow-up period was 100% complete. After the ValveClamp procedure, two patients underwent surgical MV repair or replacement, and six had recurrent residual moderate to severe MR, among whom two patients died. Mortality occurred exclusively in patients with a tenting volume index ≥0.82 mL/m^2^, as defined by 3D echocardiography. Kaplan–Meier analysis (Figure 3) revealed that patients with a tenting volume index ≥0.82 mL/m^2^ had a significantly higher probability of recurrent grade >3+ MR or re-surgery than those with a tenting volume index <0.82 mL/m^2^ (log-rank test, *P* < 0.001). ESVi ≥26.5 mL/m^2^, annular area index ≥7.24 cm^2^/m^2^, and tenting height index ≥2.37 mm/m^2^ also predicted recurrent grade >3+ MR or resurgery.

### Interobserver and Intraobserver Variabilities

The intraobserver reproducibility and interobserver reproducibility for the measurements of MVN analysis were excellent, with ICCs ranging from 0.90 to 0.97 and from 0.82 to 0.93, respectively (Table 4).

**Table 4:** Intraobserver and interobserver variabilities of MVN parameters.

**Parameters**	**Intraclass correlation coefficient (95% CI)**	
	**Intraobserver**	**Interobserver**
ALPM diameter	0.92 (0.82–0.97)	0.88 (0.78–0.97)
AP diameter	0.90 (0.81–0.97)	0.87 (0.80–0.92)
Annular area	0.97 (0.93–0.99)	0.93 (0.83–0.98)
Annular circumference	0.96 (0.92–0.98)	0.91 (0.82–0.96)
Total leaflet area	0.93 (0.83–0.97)	0.82 (0.69–0.91)
Tenting height	0.90 (0.83–0.95)	0.87 (0.74–0.96)
Tenting volume	0.96 (0.92–0.99)	0.90 (0.82–0.96)

*Abbreviations are as in Table 2*.

## Discussion

Here we present the preliminary results of a multicenter registry of a novel interventional EE device and investigation into the MV anatomic determinants of residual MR 2+ after ValveClamp implantation in patients with DMR. Our study had several notable findings: (a) at the beginning of the intervention, patients with residual MR 2+ presented with a significantly larger tenting size and MV annular diameter and area at baseline, as defined by 3D TEE imaging, among which the tenting volume index was independently associated with residual MR 2+; (b) patients with a tenting volume index ≥0.82 mL/m^2^ were more likely to have recurrent moderate to severe or greater MR during short-term follow-up; and (c) multiple clamps might be necessary for patients with a tenting volume index ≥0.82 mL/m^2^ in order to counteract more severe morphological distortion and achieve optimal results.

Many less invasive interventional treatment options for MV repair have been developed, as numerous elderly patients with symptomatic severe MR are deemed inoperable because of being high risk (18). Residual MR 2+ has been reported to be associated with recurrence of MR and lower survival rates in both surgical and interventional MV EE repair (3, 19). Our unpublished data have revealed that although ValveClamp can provide sustained and effective MR reduction as an EE-based device, some cases showed suboptimal results. With an increasing number of patients being treated, identifying MV anatomic features that could predict favorable outcomes has become an important issue.

In the current study, we identified higher sPAP, larger tenting size and annular dimension, and a more dilated LV in the residual MR 2+ group, all of which may reflect the long course of DMV disease in this group. Among these morphological changes, we demonstrated that the tenting volume index was independently associated with residual MR 2+.

MV tethering is commonly observed in patients with FMR and is negatively associated with MV coaptation (20). Otani et al. first reported primary PML prolapse in patients with DMR caused by the outward displacement of papillary muscles due to secondary LV dilatation and therefore AML tethering (7). AML tethering in patients with PML prolapse was demonstrated to be associated with unfavorable postprocedural residual MR after surgical repair (21). Moreover, regional leaflet tethering can coexist with prolapse in AML and/or PML and, more importantly, could also affect surgical repair as a risk factor for MR occurrence (10, 11, 22). Hence, tenting volume may be an integrated factor to evaluate the tethering severity of the entire MV leaflet to predict residual MR 2+ immediately after interventional EE repair. Oguz et al. analyzed 35 patients with DMR who received MitraClip implantation (10), and 13 (37%) had residual MR 2+, and univariate analysis implied that a larger tenting volume and tenting height were associated with an increased likelihood of residual MR 2+. Moreover, Kim et al. (11) reported that among 67 patients with DMR undergoing MitraClip, the LV end-diastolic diameter and mitral annular dilation augmented the risk of intraoperative residual MR 2+, highlighting the negative impact of LV and annular dilation for residual or recurrent MR; however, the tenting volume was not studied in this model. Our study adds further knowledge, as we demonstrated that the tenting volume index was the major determinant of residual MR 2+, even independent of annular dimension and ESVi.

Compared with previous MVN studies, the value of the tenting volume index for the residual MR ≤1+ group in our series is consistent with that of the healthy controls from the study by Saito et al. and the patients with DMR from the study by Oguz et al. Moreover, the value for the residual MR 2+ group (1.17 ± 0.67 mL/m^2^) is comparable with the patients with FMR (1.3 ± 0.7 mL/m^2^) from Saito et al. and patients with mixed MR (1.3–1.6 mL/m^2^) from Oguz et al. (10, 20). However, the population of our residual MR 2+ group may be different from that of the mixed/functional MR group in the study by Oguz et al., as our population presented with preserved LV function (LVEF: 50–67% vs. 30–54%) despite LV dilatation. These findings suggest that patients with DMR could evolve to mixed disease via the established vicious cycle (7, 21), where DMR causes secondary mitral leaflet tethering by LV dilatation, and leaflet tethering in turn exacerbates MR. More importantly, this subtype of MR poses a challenge to the effectiveness of interventional EE repair.

**Figure 4:**
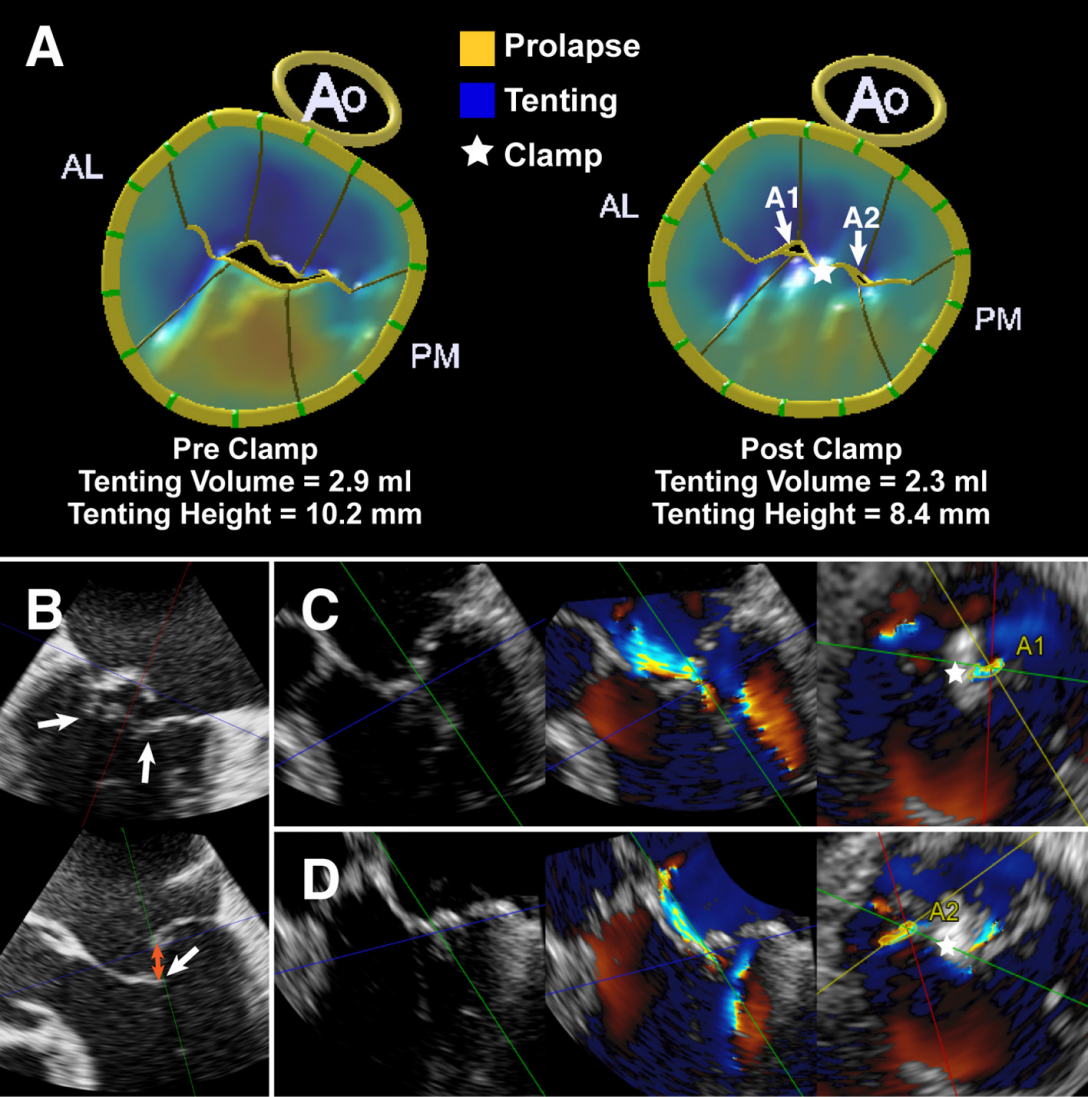
A typical case with P2 prolapse/flail and secondary bileaflet tethering. **(A)** Left: MV modeling from MVN analysis vividly demonstrating a prolapsing P2 scallop (shaded yellow) and tented scallops (shaded blue). Right: MV modeling showing the residual tenting volume (shaded blue) and residual regurgitant orifice (A1 and A2). **(B)** MV commissural view and long-axis view derived by multiplanar reconstruction (MPR) from real-time 3D dataset showing preoperative P2 prolapse/flail and leaflet tethering (white arrow). Orange arrow indicates the flail/coaptation gap. **(C,D)** MPR images derived from 3D Color Doppler dataset showing leaflet tethering with residual regurgitant jets after ValveClamp implantation. The total vena contracta area (VCA) was as follows: VCA_A1_ + VCA_A2_ = 0.16 cm^2^ + 0.12 cm^2^ = 0.28 cm^2^. Left: color compress images; Middle: color Doppler images; Right: VCA measurements. Asterisks indicate the implanted clamp.

Our study also provides new insights into the working mechanisms of EE-based devices. In our patient population, the reduction in AP diameter in the residual MR 2+ group was not statistically significant compared with the residual MR ≤1+ group immediately after ValveClamp implantation. Additionally, the residual tenting volume and annular area were more pronounced in the residual MR 2+ group than in the residual MR ≤1+ group (Figure 4). Moreover, given the observed reduction in tenting volume in the residual MR 2+ group, we speculate that the ValveClamp could improve coaptation by counteracting the augmented leaflet stress and/or remodeling strain imposed by LV and annulus dilatation (23). It can be inferred that the implantation of more clamps may reduce the tenting volume to within the normal range, leading to further AP reduction and facilitating leaflet coaptation (24, 25).

Notably, our results were derived from a population in which the majority (59/62) received implantation of only one clamp. If an aggressive strategy is adopted, the results may be different, especially for the cutoff value of the tenting volume index. Nonetheless, our study revealed that the tenting volume index was a predictor for residual MR if one clamp (or one MitraClip) was implanted and that multiple clamps (or MitraClips) might be necessary for patients with larger tenting volume indexes in order to reduce the residual MR. Among the three patients who received implantation with two clamps, one was due to a central cleft splitting the prolapsed P2 scallop with multiple regurgitant jets, and the other two cases showed a 0.84- and a 1.2-mL/m^2^ tenting volume index. All of these cases achieved mild residual MR immediately after ValveClamp implantation. However, the case with a 1.2-mL/m^2^ tenting volume index developed a leaflet tear with a 3+ residual MR at the 1-month follow-up, which may be attributable to excessive tension on the leaflet, as revealed by intraoperative TEE imaging.

We also noted a correlation between the annular area index and the tenting volume index in the residual MR 2+ group, although the leaflet–to–annulus area ratio was comparable with that of the residual MR ≤1+ group. This implies that a reliable annuloplasty may be complementary for this group to interventional EE repair to improve immediate and long-term outcomes. Further studies are needed to evaluate whether patients with DMR and increased leaflet tenting may respond more favorably to multiclamp implantation and complementary annuloplasty.

There are several inherent limitations to our study. The main limitation is the relatively small sample size. In particular, there were only 14 patients in the residual MR 2+ group; thus, multivariable results for determinants of residual MR 2+ may not be sufficiently robust, even after controlling for factor numbers. However, the current data represent the largest cohort of the ValveClamp trial to date, and the results still have a certain interpretability. Nevertheless, it is important to validate this finding in larger groups of patients with long-term follow-up. The end-systolic frame was chosen for our analysis because, at this frame, the tenting volume typically correlates with MR severity and represents the maximum prolapse size. The leaflet area was measured in end-diastole because the entire leaflet area cannot be visualized in systole. Finally, multivariate Cox regression analysis was not performed because of the low incidence of endpoint events. However, the current results are promising and have implications for subsequent interventional strategies.

## Conclusion

Preprocedural assessment of the tenting volume index will help to predict residual MR 2+ for patients with DMR who receive EE-based interventional repair. Patients with DMR with a tenting volume index ≥0.82 mL/m^2^ are more likely to have acute residual MR 2+ and recurrent moderate to severe or greater MR. Multiple clamps or more aggressive strategies might be necessary for patients with larger tenting volume indexes in order to reduce residual MR. Our observations provide important insights into patient suitability for interventional EE repair and will assist with the planning of future interventional strategies to obtain sustained optimal results for patients with DMR with unfavorable anatomy.

## Data Availability Statement

The original contributions presented in the study are included in the article/supplementary material, further inquiries can be directed to the corresponding author/s.

## Ethics Statement

The studies involving human participants were reviewed and approved by Zhongshan Hospital, Fudan University. The patients/participants provided their written informed consent to participate in this study.

## Author Contributions

ZG and WP collected data, analyzed and interpreted the results, and drafted the manuscript. WL, LW, and DK collected and analyzed the data. WP, CP, DZ, and JG contributed to the design, planning, and conduct of the study. CP, DZ, XS, and JG critically revised the manuscript. All authors contributed to the article and approved the submitted version.

## Conflict of Interest

DZ and WP are consultants for Hanyu Medical Technology. The remaining authors declare that the research was conducted in the absence of any commercial or financial relationships that could be construed as a potential conflict of interest.
